# Complete Genome Sequence of *Bacillus subtilis* GQJK2, a Plant Growth-Promoting Rhizobacterium with Antifungal Activity

**DOI:** 10.1128/genomeA.00467-17

**Published:** 2017-06-01

**Authors:** Jinjin Ma, Hu Liu, Chengqiang Wang, Zhilin Xia, Kai Liu, Qihui Hou, Yuhuan Li, Tongrui Zhang, Haide Wang, Beibei Wang, Yun Wang, Ruofei Ge, Baochao Xu, Gan Yao, Zhensheng Jiang, Wentong Hou, Yanqin Ding, Binghai Du

**Affiliations:** a College of Life Sciences/Shandong Key Laboratory of Agricultural Microbiology/National Engineering Laboratory for Efficient Utilization of Soil and Fertilizer Resources, Shandong Agricultural University, Tai’an, China; b Zunyi Tobacco Monopoly Administration of Guizhou, Zunyi, China; c College of Resources and Environment, Shandong Agricultural University, Tai’an, China; d State Key Laboratory of Nutrition Resources Integrated Utilization, Linshu, China

## Abstract

*Bacillus subtilis* GQJK2 is a plant growth-promoting rhizobacterium with antifungal activity which was isolated from *Lycium barbarum* L. rhizosphere. Here, we report the complete genome sequence of *B. subtilis* GQJK2. Ten gene clusters involved in the biosynthesis of antagonistic compounds were predicted.

## GENOME ANNOUNCEMENT

*Bacillus subtilis* is a model species of the *Bacillus* genus and is widely used in scientific research. It has also been applied extensively in agricultural production for its important role in controlling some plant pathogens by producing surfactin (1), iturin A (2), fengycin (3), macrolactin N (4), and difficidin (5). In addition, some other mechanisms promoting plant growth exist in *B. subtilis*, such as indole-3-acetic acid (IAA) production (6), siderophore production (7), and phosphate solubilization (8). *B. subtilis* GQJK2 was isolated from the rhizosphere of *Lycium barbarum* L. in Ningxia, China, and was identified to effectively inhibit the pathogen *Fusarium solani*, which can cause root rot of *Lycium barbarum* L.

The complete genome of* B. subtilis* GQJK2 was sequenced by the Illumina HiSeq and PacBio platforms. A total of 1,017 Mb of clean raw data were generated by the HiSeq platform, and the genome coverage was 278.0×. Meanwhile, 124,214 subreads of about 1,253,008,644 bp were obtained through PacBio. SMRT Analysis 2.3.0 (9) (https://github.com/PacificBiosciences/SMRT-Analysis/wiki/SMRT-Pipe-Reference-Guide-v2.3.0) was used to assemble the sequence. The genome annotation was carried out by the NCBI Prokaryotic Genome Annotation Pipeline (PGAP) (https://www.ncbi.nlm.nih.gov/genome/annotation_prok/). The carbohydrate-active enzymes were analyzed by CAZy version 20161020 (10) (http://www.cazy.org/). The secondary metabolism clusters were determined with antiSMASH version 3.0.5 (11).

*B. subtilis* GQJK2 contains a 4,072,961-bp circular chromosome with a G+C content of 43.76%. A total of 4,190 genes were annotated, including 3,976 coding genes, 30 rRNA genes, 86 tRNA genes, 5 noncoding RNA (ncRNA) genes, and 93 pseudogenes. The carbohydrate-active enzymes were encoded by 163 genes, among which 40 genes were relevant to carbohydrate-binding modules (CBMs), 55 genes could encode glycoside hydrolases (GHs), 42 genes were germane to glycosyl transferases (GTs), and 26 genes were involved in carbohydrate esterases (CEs), polysaccharide lyases (PLs), or auxiliary activities (AAs). Ten gene clusters relating to secondary metabolism biosynthesis were predicted. Two of them showed high similarity with gene clusters that were previously reported. One gene cluster (BSK2_09705 to BSK2_09920) belonging to nonribosomal peptide synthetase (Nrps) type showed 100% similarity to the fengycin biosynthetic gene. The other gene cluster (BSK2_19145 to BSK2_19345) was comparable to the bacilysin biosynthetic gene cluster. The other gene clusters might produce surfactin, bacillaene, bacillibactin, subtilosin_A, terpene, and type 3 polyketide synthase (T3pks). The genome sequence of *B. subtilis* GQJK2 and its annotation further present the probable molecular genetic characteristics of *B. subtilis* and are aso beneficial for its application in agricultural production.

### Accession number(s).

The whole-genome sequence of *B. subtilis* GQJK2 has been deposited at GenBank under accession number CP020367.
